# Exploring the Dose–Effect Relationship of *Bifidobacterium longum* in Relieving Loperamide Hydrochloride-Induced Constipation in Rats through Colon-Released Capsules

**DOI:** 10.3390/ijms24076585

**Published:** 2023-04-01

**Authors:** Xin Zhou, Bingyong Mao, Xin Tang, Qiuxiang Zhang, Jianxin Zhao, Hao Zhang, Shumao Cui

**Affiliations:** 1 State Key Laboratory of Food Science and Technology, Jiangnan University, Wuxi 214122, China; 2 School of Food Science and Technology, Jiangnan University, Wuxi 214122, China; 3 National Engineering Research Center for Functional Food, Jiangnan University, Wuxi 214122, China

**Keywords:** *Bifidobacterium longum*, colon-released capsules, constipation, dose–effect relationship

## Abstract

Constipation is a common disease affecting humans. *Bifidobacterium longum* is reportedly effective in relieving constipation. Current studies generally focus on the dose–response relationship of oral doses; however, the dose–effect relationship of *B. longum* in the colon, which is the primary site where *B. longum* exerts constipation-relieving effects, to treat constipation has not been studied. Herein, three strains of *B. longum* (FGSZY6M4, FJSWXJ10M2, and FSDJN6M3) were packaged in colon-released capsules to explore the dose–effect relationship in the colon. For each strain, three groups of capsules (10^4^, 10^6^, and 10^8^ CFU/capsule, respectively) and one group of free probiotics (10^8^ CFU/mL) were used to explore the colonic dose effect of *B. longum*. The results showed that the three strains of *B. longum* improved fecal water content and promoted intestinal motility by regulating gastrointestinal peptide (MTL, GAS, and VIP), aquaporin-3, and 5-hydroxytryptamine levels while promoting gastrointestinal motility and relieving constipation by regulating the intestinal flora composition of constipated rats and changing their metabolite content (short-chain fatty acids). Among the three free bacterial solution groups (10^8^ CFU/mL), FGSZY6M4 was the most effective in relieving constipation caused by loperamide hydrochloride in rats. The optimal effective dose of each strain was 6M4 (10^4^ CFU/day), 10M2 (10^6^ CFU/day), and S3 (10^8^ CFU/day) of the colon-released capsules. Therefore, for some effective strains, the dose of oral probiotics can be reduced by colon-released capsules, and constipation can be relieved without administering a great number of bacterial solutions. Therefore, investigating the most effective dose of *B. longum* at the colon site can help to improve the efficiency of relieving constipation.

## 1. Introduction

With the accelerating pace of life, constipation has become a common disease of the digestive system due to the influence of age, diet, and pressure, among other factors [1,2,3]. Furthermore, women and the elderly are more likely to suffer from constipation [4], which may be because that women are more susceptible to stress or pregnancy, and the elderly have decreased gastrointestinal motility and limited diet and are affected by diseases and drugs. The number of constipation cases has reached 30% in European and American countries [2] and continues to grow in China. According to the American Gastroenterological Association, patients with constipation can be classified into three types: normal-transit constipation, slow-transit constipation, and defecation disorders [5]. Dietary fiber supplements [6] and laxatives are common therapeutic options [7]. However, dietary fiber supplementation takes a long time and laxatives such as polyethylene glycol, lactulose, bisacodyl, and sodium picosulfate may cause side effects and result in drug resistance [8,9,10]. The use of laxatives may result in bloating, colic, melanosis coli, and electrolyte disturbances and, in some trials [8,11,12], may also increase the risk of death from ischemic stroke or coronary heart disease [13,14].

According to an increasing body of research, probiotics can relieve constipation by regulating intestinal flora, improving bowel rating, and promoting intestinal movement. Owing to their safety and the absence of side effects compared with conventional laxatives, probiotics are regarded as good substitutes [15]. *Bifidobacterium* spp., such as *Bifidobacterium animalis*, *Bifidobacterium longum*, and *Bifidobacterium bifidum*, in particular, have been shown by many studies to play a key role in relieving constipation [16,17]. The mechanisms by which probiotics relieve constipation can be summarized as follows: firstly, individuals with constipation experience changes in their intestinal flora, mostly characterized by a decline in the quantity of Bacteroidetes, an increase in the abundance of *Firmicutes*, and, notably, a considerable decline in the abundance of *Bifidobacterium* [18,19]. Bifidobacterium supplementation is recommended in constipation patients to properly restore the intestinal ecological balance. Secondly, short-chain fatty acids (SCFAs) and other metabolites produced by the intestinal flora have an impact on intestinal motility [20]. SCFAs can stimulate mucosal receptors linked to the intestinal or vagus nerves [21], or they can act directly on the smooth muscle of the colon [22], thereby altering intestinal motility [23,24]. Additionally, Wang et al. discovered that intragastric treatment with *B. longum* in mice with constipation may considerably enhance the quantity of acetic acid and propionic acid in stools [25]. Finally, *B. longum* can effectively regulate the level of gastrointestinal active peptides, the expression of 5-hydroxytryptamine (5-HT, also known as serotonin), and its receptor, as well as the expression of water channel proteins in serum [25]. Chai discovered that the gastrointestinal active peptide level changed and the expressions of 5-HT and 5-HT receptor 4 (5-HT4R) were promoted in constipated mice after 38 days of *B. longum* intervention, but it did not affect the level of SCFAs [26].

Studies on the dose–effect relationship in the colon of *B*. *longum* in treating constipation are currently scarce. Probiotics are generally considered effective at oral doses greater than 10^6^ CFU, and experiments most frequently use intragastric doses of 10^8^–10^10^ CFU/mL [27]. *Bifidobacterium* is sensitive to an environment with gastric acid and bile salt; therefore, after passing through the stomach and upper part of the small intestine, its ability to survive is drastically reduced. As a result, the quantity and vitality of microorganisms entering the intestine decrease substantially, affecting their ability to regulate intestinal microecology [28]. Therefore, the number of probiotics that enter the colon remains unknown.

To investigate the dose–effect relationship of *B. longum* in treating constipation in rats, colon-released capsules were used to package the bacteria at different doses (10^4^, 10^6^, and 10^8^ CFU/capsule) and compared to free bacterial suspension (10^8^ CFU/mL). The first black stool defecation time, propulsion rate, and fecal water content were measured to detect constipation in rats. The gastrointestinal active peptides MTL, GAS, and VIP were used to monitor intestinal motility, and 5-HT and aquaporin-3 (AQP3) were used to assess intestinal motility and water absorption. Additionally, the intestinal flora distribution and fecal SCFAs were measured to assess whether there was a dose–effect link between each dose group.

## 2. Results

### 2.1. Viable Number of Bifidobacterium longum in Capsules

After encapsulation, *B. longum* was stored in a refrigerator at 4 °C and counted before use. The free bacterial solution group was re-dissolved with normal saline before intragastric injection and counted (after counting, there was no notable change in the number of viable bacteria within 3 weeks) to achieve the necessary concentration (more than 5 × 10^8^ CFU/mL). Table 1 presents the results.

### 2.2. IVIS Imaging Shows That the Capsule Was Released at the Colon Site

**Figure 1:**
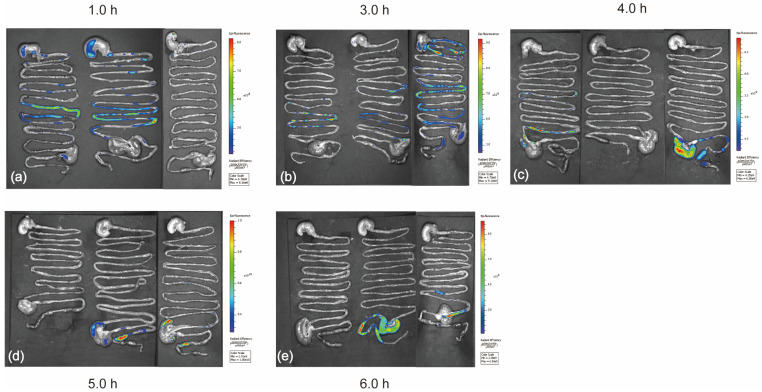
Intestinal fluorescence of rats indicating capsule transport. (**a**–**e**) The gastrointestinal fluorescent images of rats at different time points. The red color indicates a higher fluorescence signal, and the blue color indicates a lower fluorescence signal.

Rats have thicker skin layers than mice, which may block the fluorescence signal. Therefore, the rats were dissected at fixed time points, and fluorescence photographs were taken directly of their gastrointestinal tract. The fluorescence images obtained using the IVIS system are shown in Figure 1. It can be seen that the fluorescence capsule was transported to the colon in approximately 5–6 h, according to the fluorescence distribution shown in Figure 1d,e. The fluorescence remained aggregated and did not diffuse, indicating that the capsule maintained its shape in the stomach and small intestine. No disintegration or leakage of fluorescent material was observed. Therefore, the capsule could effectively achieve precise release at the colon site.

### 2.3. B. longum Can Effectively Relieve the Apparent Symptoms of Constipation

**Figure 2:**
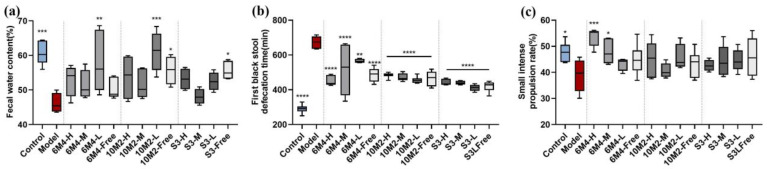
Apparent indicators of constipation: (**a**) fecal water content (%); (**b**) first black stool defecation time (min); (**c**) small intestine propulsion rate (%). Data are shown as mean ± SD (*n* = 6). * *p* < 0.05, ** *p* < 0.01, *** *p* < 0.001, **** *p* < 0.0001, compared with the model group.

After 21 days of probiotic intervention and 14 days of loperamide hydrochloride administration, the constipation model was preliminarily determined by observing the apparent indices of rats. The results and significance (one-way ANOVA, *p* < 0.05) of fecal water content, first black stool defecation time, and small intestine propulsion rate are shown in Figure 2. The control and model groups showed significant differences (*p* < 0.05) in three apparent indices: the fecal water content of the control group was significantly higher, the time of the first black stool was significantly shorter, and the intestinal propulsion rate was higher than those of the model group.

The fecal water content (Figure 2a) indicates the dryness of the rat stool, which is a characteristic indicator of constipation. The fecal water content in the probiotic intervention group was higher than that in the model group, and the fecal water content in the 6M4-L and 10M2-L capsule groups was higher than that in the model group, which was similar to that in the control group. The fecal water content in the 10M2-Free and S3-Free groups in the free bacterial solution group also greatly increased after constipation. Although the water content of the medium- and high-dose capsule groups was slightly higher than that of the model group, the difference was not statistically significant (*p* > 0.05). In conclusion, administration of *Bifidobacterium longum* microcapsules or free bacterial solution can increase fecal water content and relieve constipation caused by loperamide hydrochloride.

The first black stool time (Figure 2b) was recorded from the time of intake of the carbon meal (one hour after oral administration of loperamide hydrochloride) to the time of the first collection of stools containing black ink, reflecting the transport ability of the entire gastrointestinal tract. The average time of the first black stool in the model group was approximately 671 min, which was much later than that of the control group (290 min) (*p* < 0.001), indicating that rats administered loperamide hydrochloride gavage showed symptoms of considerably prolonged defecation time. This is the main manifestation of constipation. As for the groups of capsules and free bacterial solution, the first black stool defecation time was significantly shorter than that in the model group (*p* < 0.05) but slightly longer than that in the control group, which indicates that intragastric *B. longum* can effectively promote gastrointestinal motility and shorten gastrointestinal transportation time.

The propulsion rates of the small intestine (Figure 2c) were measured by calculating the distance traveled by the carbon meal as a proportion of the entire small intestine 30 min after intragastric administration. The propulsion rate of the control group was higher than that of the model group, but the difference was not particularly significant, and the propulsion rate of each intervention group was also slightly higher than that of the model group, showing *p* > 0.05. There are two speculations regarding these results. Thirty minutes is not sufficient to observe a significant difference. However, because 12 h of fasting were performed before the intragastric carbon diet, this speculation is not based on evidence. Another reasonable hypothesis is that the main role of *B. longum* in alleviating constipation in rats is not in the small intestine and that its effective position may be in the colon, where the flora is abundant.

### 2.4. B. longum Exhibits a Dose–Effect Relationship in the Regulation of Gastrointestinal Active Peptides in Constipated Rats

**Figure 3:**
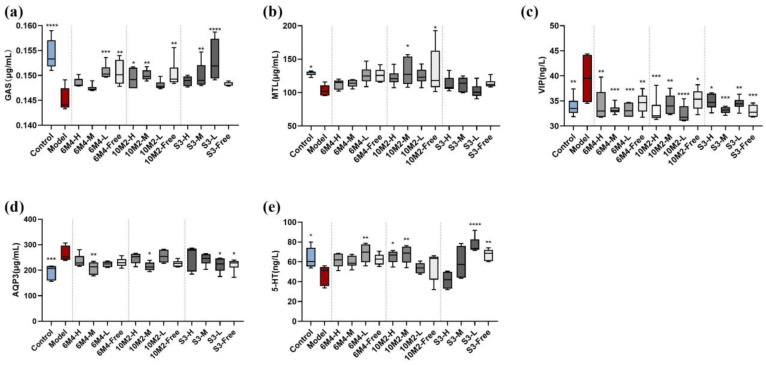
Serum gastrointestinal active peptide levels (**a**–**c**), colonic aquaporin-3, and serotonin levels. (**a**) Serum gastrin (GAS); (**b**) serum motilin (MTL); (**c**) serum vasoactive intestinal peptide (VIP); (**d**) colonic aquaporin-3 (AQP3); (**e**) colonic serotonin (5-HT). Data are shown as mean ± SD (*n* = 6). * *p* < 0.05, ** *p* < 0.01, *** *p* < 0.001, **** *p* < 0.0001, compared with the model group.

Serum levels of gastrointestinal active peptides determined by ELISA are shown in Figure 3, including excitatory gastrointestinal active peptides MTL and GAS, and inhibitory gastrointestinal active peptide VIP. The MTL and GAS levels in the model group (MTL, *p* < 0.05; GAS, *p* < 0.0001) were significantly lower than those in the control group, and the VIP (*p* < 0.01) levels were significantly higher than those in the control group. All probiotic intervention groups had appropriately increased levels of excitatory gastrointestinal active peptide, and all three strains could significantly increase the GAS level in constipated rats. Although the serum MTL level in each group was higher than that in the model group, only the 10M2 group showed significantly increased MTL levels (*p* < 0.05). The VIP level of each intervention group was significantly lower than that of the model group (*p* < 0.05), and the effect was similar to that of the control group.

### 2.5. B. longum Can Decrease the Content of Aquaporin-3 and Increase Serotonin Content in Colon Tissue to Promote Intestinal Peristalsis

AQP3 is involved in water absorption in the colon and is closely associated with constipation. As shown in Figure 3d, serum AQP3 levels in the model group were significantly higher than those in the control group (*p* < 0.05). The level of AQP3 in each group administered *B. longum* was lower than that in the model group, and the levels in the 6M4-M (*p* < 0.01), 10M2-M (*p* < 0.05), S3-L (*p* < 0.05), and S3-Free (*p* < 0.05) groups were significantly lower than those in the model group, making the serum AQP3 level close to that of the blank control group.

The colonic serotonin levels in the model group were significantly lower than those in the control group, according to Figure 3e, and all the intervention groups of the constipated rats had improved 5-HT levels and they were restored to the 5-HT levels of the healthy rats in the control group. The serotonin levels in the colon were significantly increased in the 6M4-L (*p* < 0.01), 10M2-H (*p* < 0.05), 10M2-M (*p* < 0.01), and S3-L (*p* < 0.0001) groups.

### 2.6. B. longum Can Increase the Content of SCFAs in Feces to Promote Intestinal Motility

**Figure 4:**
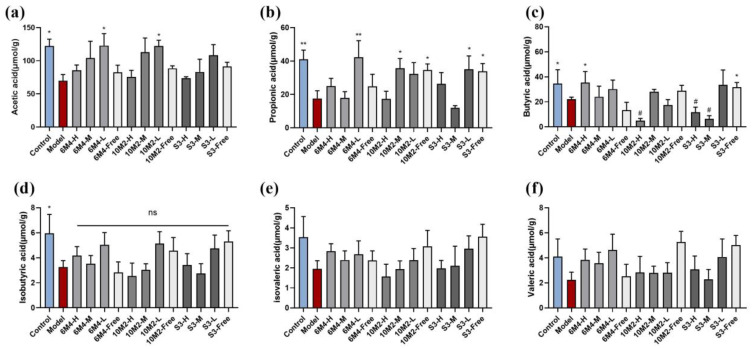
The short-chain fatty acid level of fecal samples by GC-MS. (**a**) Acetic acid (μmol/g); (**b**) propionic acid (μmol/g); (**c**) butyric acid (μmol/g); (**d**) isobutyric acid (μmol/g); (**e**) isovaleric acid (μmol/g); (**f**) valeric acid (μmol/g). Data are shown as mean ± SD (*n* = 6). * *p* < 0.05, ** *p* < 0.01, compared with the model group. # *p* < 0.05, compared with the control group. ns, not significant.

The fecal SCFA content is depicted in Figure 4, including acetic acid, propionic acid, butyric acid, isobutyric acid, isovaleric acid, and valeric acid. Levels of all types of SCFA in the model group were lower than those in the control group. This suggests that rats with constipation have much lower intestinal SFCA content. According to these findings, only acetic acid, propionic acid, and butyric acid showed substantial changes (*p* < 0.05). When compared to the model group, the concentrations of isobutyric acid, isovaleric acid, and valeric acid in the treatment groups did not change appreciably.

A linear relationship between acetic acid concentration and SCFA content (Figure 4a) was particularly fascinating, and the concentration increased as the dose decreased in the capsule group within the same strain. This demonstrates that *B. longum* at 10^4^ CFU/capsule has a greater impact on the colon than live bacteria at 10^8^ CFU/capsule, which merits further discussion.

As shown in Figure 4b, the fecal propionic acid content increased considerably in all three low-dose capsule groups, reaching a level comparable to that of the blank group. Additionally, in the 10M2 group, there was no correlation between propionic acid concentration and dose, indicating that 10M2 may have a greater capacity to encourage gut flora to produce propionic acid, regardless of dose.

The butyric acid content in feces is shown in Figure 4c, and the three capsule groups of 6M4 all showed increased butyric acid levels in constipated rats compared with that in the model group, especially 6M4-L (*p* < 0.05). However, the 10M2-H and S3-H groups showed lower butyric acid content than the model groups, 10M2-L and S3-L, which may indicate that the release of high doses of probiotics in the colon may affect bacterial metabolism, possibly by inhibiting the production of butyric acid from other bacteria.

### 2.7. Gut Microbiota

**Figure 5:**
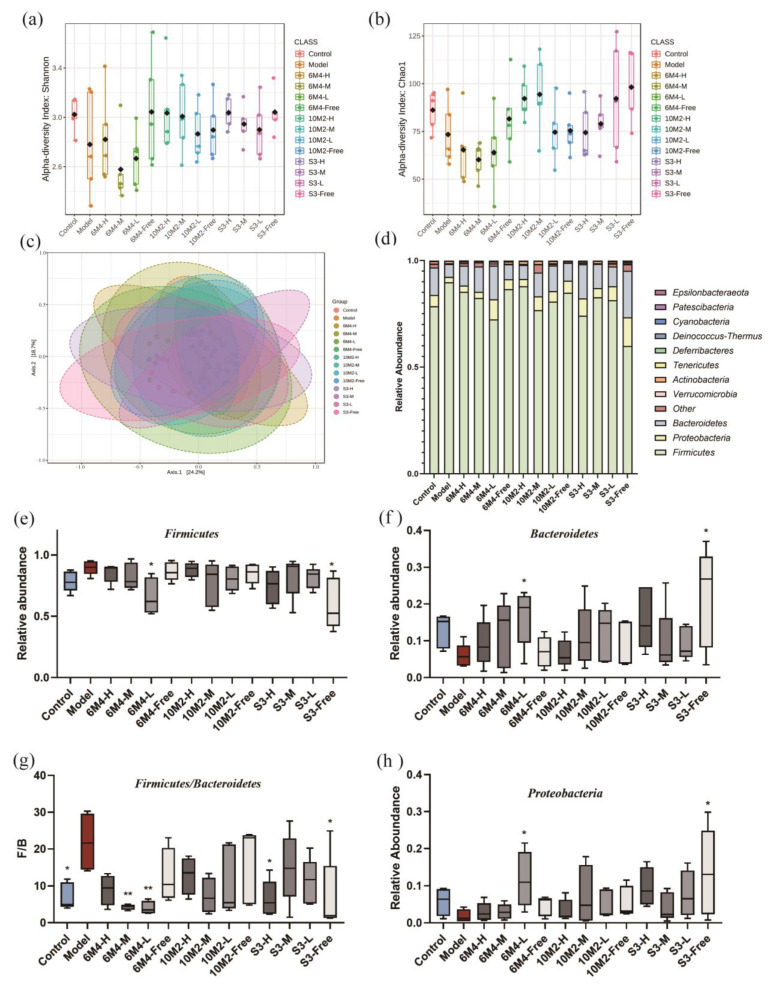
The composition of intestinal flora was changed after being given different doses of *B*. *longum*. (**a**) Shannon index of α-diversity; (**b**) Chao1 index of α-diversity; (**c**) principal coordinate analysis (PCoA) of the microbial group indicating β-diversity; (**d**) relative abundance of phyla; (**e**–**h**) relative abundance of Firmicutes, Bacteroidetes, Firmicutes/Bacteroidetes, and Actinomyces. Data are shown as means ± standard deviations. * *p* < 0.05, ** *p* < 0.01, compared with the model group.

*Bifidobacterium longum* can relieve constipation via different mechanisms by adjusting the composition of the intestinal flora. Therefore, we analyzed the fecal flora composition of the rats. The Shannon and Chao1 indices of rats in *B. longum* intervention groups were higher than those in the model group, indicating that *B. longum* intervention successfully reversed the loperamide hydrochloride-induced α-diversity loss. PCoA showed a distinct cluster of microbiota compositions among the groups (Figure 5c). The intervention group showed more overlap with the control group, suggesting that *Bifidobacterium longum* had a notable effect on the transformation of intestinal microbial composition from constipated rats to normal rats.

The phylum-level analysis of the intestinal flora of rats (Figure 5d) revealed that the gut flora of rats primarily consists of Firmicutes, Proteobacteria, Bacteroidetes, Verrucobacteria, and Actinomyces. The ratios of Firmicutes and Bacteroidetes (F/B) are the principal microbiota linked with constipation, and the results are shown in Figure 5e–g. Each *B. longum* intervention group had an increased relative abundance of Bacteroidetes while the relative abundance of Firmicutes was decreased in constipated rats. The F/B ratios of the 6M4-M, 6M4-L, S3-H, and S3-Free groups decreased significantly compared to those of the model group. Moreover, the relative abundances of Proteobacteria in the 6M4-L and S3-Free groups were notably higher than those in the model group.

**Figure 6:**
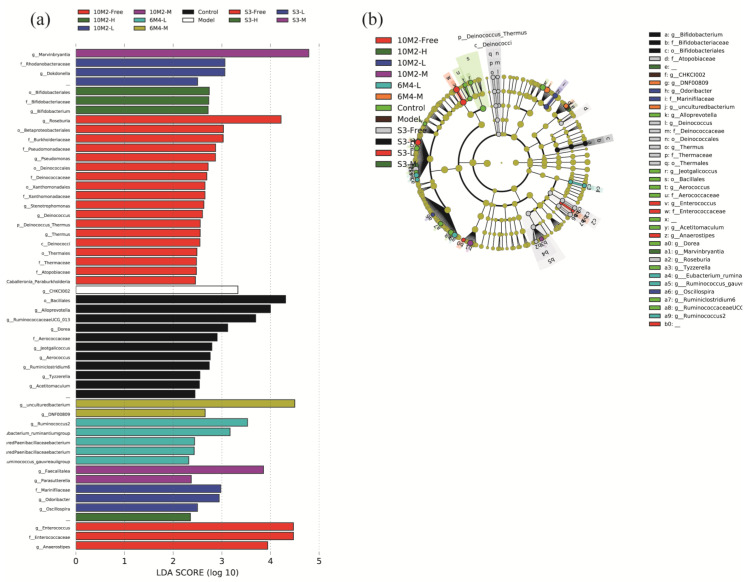
The linear discriminant analysis effect size (LEfSe) analysis of fecal flora. (**a**) LDA score of characteristic bacteria (LDA > 2); (**b**) phylogenetic tree illustrating the taxonomic hierarchy of bacteria, which varies in terms of relative abundance between the various groupings. The relative abundance of each taxon is proportional to the size of the circle.

To further investigate the variations in intestinal flora distribution, LEfSe was used to calculate the LDA score (>2) for relative abundance at the genus level in each group (Figure 6). As we can see, the characteristic bacteria, Alloprevotella, in the model group belong to the phylum Actinobacteria. The most abundant genus in the control group belonged to Bacteroidetes, whereas the other characteristic genera were all from Firmicutes. Firmicutes and Actinobacteria were the two most abundant taxa in the 6M4-M group. The most abundant taxa in the 6M4-L group were the genus *Ruminococcus*, two from the phylum Firmicutes, and some other key genera from the phylum Tenericutes. The most abundant taxa of 10M2-H, 10M2-M, and 10M2-Free belonged to Firmicutes, while the key bacteria of 10M2-L belonged to Bacteroidetes. The key genera of S3-Free and S3-M belong to Firmicutes, while those of S3-H and S3-L belong to Bacteroidetes, which is also consistent with the result of reducing the F/B ratio.

## 3. Discussion

Constipation is a prevalent disease worldwide. The conventional method of treating constipation involves the use of laxatives and other drugs; however, these drugs can have negative effects over time. Therefore, the development of innovative techniques is critical. According to recent animal experiments and clinical population experiments, probiotics can effectively regulate intestinal flora and achieve constipation relief without the side effects of drug treatment; therefore, they are considered the recommended therapy for constipation [29].

According to a meta-analysis, the majority of dosages used in animal trials and clinical investigations ranged from 10^6^ to 10^9^ CFU/day, but the response to doses varied between strains [27]. According to a study by Whorwell et al., *B. infantilum* 35624 was only helpful in lowering irritable bowel syndrome in adult women at 10^8^ CFU/day, with no discernible difference between 10^6^ and 10^10^ CFU/day and placebo [30]. Additionally, a population study by Larsen et al. revealed that, as the dose increased, the fecal recovery of BB-12 considerably increased. There was a substantial linear increase in fecal consistency (looser stool) with increasing probiotic dose (10^8^, 10^9^, 10^10^, and 10^11^ CFU/day) [31]. However, all of the above studies explored the dose–effect relationship of direct oral administration of probiotics to relieve constipation but did not consider the actual number of live bacteria effective in the colon. Thus, to investigate the colonic dose–effect connection of *B*. *longum* in treating constipation, we creatively adopted a capsule that can prevent the release of probiotics in the colon and designed 10^4^, 10^6^, and 10^8^ CFU/capsule *B*. *longum* and 10^8^ CFU/mL free bacterial suspension groups.

We found that all three strains could relieve constipation to various degrees and that different doses presented different relieving effects. From the results of the apparent indicators, the greater effect of the free bacterial solution group with the same dose on fecal water content was seen with 6M4 and 10M2, and S3 had a poor effect. The time of the first black stool was significantly earlier, indicating that administration of *B*. *longum* can effectively promote defecation and may have a significant effect on promoting gastrointestinal motility. There was no significant difference in the propulsion rate of the small intestine; therefore, it was inferred that *B*. *longum* was mainly concentrated in the colon rather than the small intestine.

Rats with constipation also frequently have poor gastrointestinal motility, which may be reflected in the level of gastrointestinal active peptides [32,33]. There are two categories of digestive active peptides: excitatory and inhibitory. Three types of gastrointestinal peptides were used in this study. MTL and GAS are excitatory, and VIP is inhibitory. MTL can stimulate pepsin production and duodenal contraction, both of which help in gastrointestinal peristalsis [34,35]. GAS helps to encourage pyloric sphincter relaxation and gastrointestinal smooth muscle contraction. VIP is a vasoactive intestinal peptide that helps relax smooth muscle [36]. A growing body of evidence points to the role of gut flora in reducing constipation caused by loperamide hydrochloride via these gastrointestinal peptides [37]. We also found that the three strains had different regulatory effects on active gastrointestinal peptides in constipated rats. 6M4 and S3 primarily act on GAS and VIP, and 10M2 mostly acts on MTL and VIP. This suggests that various *Bifidobacterium* strains have different mechanisms for relieving constipation. In terms of dose, 6M4-L showed a better ability to control gastrointestinal peptides than 6M4-H and 6M4-M. This shows that administration of *B*. *longum* at high concentrations directly to the colon might be useless. The results showed that the 10M2 strain regulated gastrointestinal active peptides most effectively when the colon dose reached 10^6^ CFU, encouraging intestinal peristalsis and fecal excretion.

Additionally, constipation frequently decreases the water content of stools, leaving them dry, hard, and challenging to pass. AQP3 is a crucial functional molecule for water transport in the colon and is mostly expressed in mucosal epithelial cells [38,39,40]. Loperamide hydrochloride dramatically increases the amount of AQP3 in rat colons, causing water to flow from the side of the cavity to the side of the blood vessel and harden stool [41]. *B. longum* treatment significantly decreased AQP3 levels, reduced water transfer, and prevented fecal sclerosis in the 6M4-L, S3-L, and 6M4-M groups.

Most (95%) 5-HT, also known as serotonin, which is associated with gastrointestinal motility, is released by enterochromaffin cells (ECs) [42,43]. According to a previous study, *Clostridium* can control 5-HT signaling by generating soluble metabolites that affect platelet aggregation and gastrointestinal motility [19]. In rats with constipation receiving fecal transplantation, Cao et al. discovered that 5-HT content was lowered and gastrointestinal transport time was prolonged [44]. In this study, the colonic 5-HT expression level in the model group was notably lower than that in the control group.

In accordance with data on fecal water content, prior research has demonstrated that an increase in intestinal SCFA content causes an increase in intestinal osmotic pressure, which in turn increases the intestinal lumen water content (Figure 2). The 5-HT levels herein are compatible with another hypothesis from a previous study that SCFAs can stimulate 5-HT receptors, causing ECs to produce 5-HT and speed up colonic transit [45]. In addition, acetic acid, propionic acid, and butyric acid showed significant differences in SCFA content (Figure 4).

Combined with the results in Figure 6, it was discovered that the majority of the distinctive bacterial species in the *B*. *longum* intervention group were SCFA-producing bacteria (mainly concentrated in Bifidobacteriaceae, Ruminococcaceae, and Lachnospiraceae) [46,47,48]. They also play crucial roles in the control of gastrointestinal motility. Acetic acid and butyric acid are among the metabolites produced by the main bacterial genera of 6M4-L, which are from Ruminococcaceae and Lachnospiraceae, and are in line with the fecal SCFA composition of the group. According to a previous report, Ruminococcaceae is the primary producer of butyric acid and can create acetic and propionic acids [49]. Acetic acid and butyric acid are the primary products of Lachnospiraceae bacterial metabolism [50]. Therefore, *B*. *longum* consumption can enhance the amounts of acetic acid, propionic acid, and butyric acid in constipated rats, probably because of the abundance of the above SCFA-producing bacteria in the intestinal tract [51]. *Faecalitalea* and *Parasutterella* are distinctive bacterial genera of 10M2-M, and the latter is associated with bile acid secretion in the gastrointestinal tract [52]. The *Pseudomonadaceae* family includes distinctive bacteria responsible for 10M2-H and 10M2-Free, which mostly create acetic acid. *Bifidobacterium*, a bacterium that can produce both lactic acid and acetic acid [53], is a typical bacterium of S3-H. The characteristic bacterium of S3-M, Marvinbryantia, also belongs to the family Lachnospiraceae.

In summary, different *B. longum* strains relieve constipation via different mechanisms and show the best results at specific doses. By increasing GAS secretion and lowering VIP levels, 6M4 and 10M2 increase intestinal motility. 10M2 stimulates intestinal motility by increasing MTL secretion and decreasing VIP. All three strains were able to increase fecal water content by lowering AQP3 expression and increasing intestinal water levels. The results of fecal SCFAs and horizontal flora analysis showed that the three strains could promote the secretion of SCFAs (acetic acid, propionic acid, and butyric acid) by regulating the intestinal flora composition of constipated rats, thereby improving the level of 5-HT and improving gastrointestinal motility to relieve constipation. Of the three doses of the three strains, 6M4-L, 10M2-M, and S3-M were the most effective in relieving constipation. In other words, the best colonic dose for 6M4 was 10^4^ CFU/day, while the best colonic doses for 10M2 and S3 were 10^6^ CFU/day and 10^8^ CFU/day, respectively, suggesting that different strains had an optimal dose for relieving constipation.

## 4. Materials and Methods

### 4.1. Strain Preparation

Three strains of *B. longum* (FGSZY6M4, FJSWXJ10M2, and FSDJN6M3, referred to as 6M4, 10M2, and S3, respectively) were selected from the Culture Collection of Food Microorganisms at Jiangnan University. Among the three strains, FGSZY6M4 had been registered as a patent strain CCFM1114 in Chinese patent (ZL113943682A), and could be regarded as standard reference. The strains were separated and purified on MRS plates at 37 °C using an anaerobic incubator. After three rounds of activation, the fermentation solution was collected and centrifuged (8000× *g*, 15 min, and 4 °C). Then, the bacterial sludge was collected and mixed with freeze-dried protectants at a mass ratio of 1:1. Finally, the mixture was freeze-dried in a vacuum freeze-dryer. The resulting powder was placed in a light-proof aluminum foil bag for capsule preparation. The preparation of freeze-dried protectants was as follows: 15% sorbitol and 5% glycine (Sinopharm Chemical Reagent Co., Ltd., Shanghai, China) were sterilized by high-pressure steam at 121 °C for 15 min after distillation and then used after cooling.

### 4.2. Encapsulation of Bifidobacterium longum Capsules

The oral colonic location-based drug delivery system (OCDDS) was independently developed by Nanjing Healsoul Biological Technology Co., Ltd. (Nanjing, China). The system can load live bacterial drugs into colonic location-based drug-release capsules. Through oral delivery, live bacteria can be transported directly to the colon of patients and released after avoiding the release of live bacterial drugs in the stomach, duodenum, jejunum, and ileum to initiate their therapeutic or other effects.

Capsule preparation can be summarized as follows: 0.01 g of freeze-dried *B. longum* powder was weighed and sealed in the OCDDS capsule. The capsule was a cylindrical container with one end open, which should be sealed after loading the bacterial powder. The above operations were performed on a super-clean table. The capsules were cylindrical in shape and approximately 5 mm long and 1.5 mm in diameter; these were fed to rats using a specially made gavage needle. To verify the number of viable bacteria in the capsules, three were randomly selected, and the bacterial powder in the capsules was counted on a plate using the gradient dilution method to ensure that the three doses of each strain were 10^4^, 10^6^, and 10^8^ CFU/capsule. In the following text, the three doses are represented by low, medium, and high (L, M, and H, respectively).

### 4.3. Colonic Release Validation of Capsules

Fluorescein isothiocyanate (FITC; J&K Scientific Ltd., Shanghai, China) was used as the fluorescent substance for the capsule intestinal tracer [54]. Each capsule was packed with 0.01 g of FITC powder. Fifteen normal-feeding healthy Sprague Dawley (S-D) rats were divided into three groups, and the above capsules containing fluorescence were administered according to the time point, one capsule per rat, and the time of gavage was recorded. In vivo imaging was performed at different time points (1, 3, 4, 5, and 6 h), and three rats were set apart as parallels at each time point. After reaching the corresponding time point, the rats were anesthetized and dissected. The digestive tract from the stomach to the colon was completely removed, neatly placed on paper with a black background, and placed in an in vivo imaging system (IVIS Lumina XRMS Series III, PerkinElmer, Waltham, MA, USA) for fluorescence photography [55].

### 4.4. Animal Experimental Design

**Figure 7:**
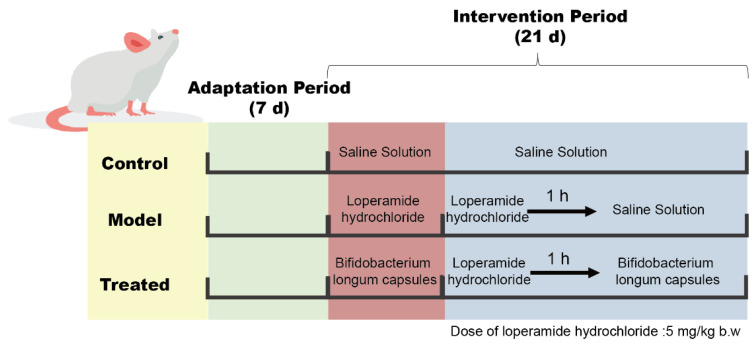
Animal experiment design schematic.

Eighty-four 6-week-old male specific pathogen-free S-D rats were purchased from Beijing Vital River Laboratory Animal Technology Co., Ltd. (Beijing, China). The breeding room was maintained at a temperature of 25 ± 2 ℃, constant humidity of 50 ± 5%, and a 12 h light–dark cycle. After a week of adaptive feeding, the animal experiment began, and all animals were fed regular food and had free access to water. The grouping information is listed in Table 2. After a week of adaptive feeding, the control and model groups were given 0.2 mL of normal saline by gavage. The remaining groups were administered corresponding doses of capsules or suspensions containing bacteria. After 3 weeks, the rats in the control group were still administered with normal saline, whereas those in the other groups were administered with 0.2 mL of loperamide hydrochloride (Xian Janssen Pharmaceutical Co., Ltd., Xi’an, China) dissolved in normal saline (5 mg/kg bw). One hour later, those other groups continued to receive the corresponding doses of capsules or suspensions containing bacteria for another 7 d. The timeline is shown in Figure 7. All animal procedures were performed in accordance with the Guidelines for Care and Use of Laboratory Animals of Jiangnan University (SYXK 2021-0045) and were approved by the Animal Ethics Committee of Experimental Animals at Jiangnan University (approval number: JN. No. 20210630S0960905) [224].

#### 4.4.1. Water Content of Feces

Fecal samples were collected from each rat and added to 1.5 mL Eppendorf tubes. The total fecal weight was immediately recorded as the wet weight. After lyophilization, the fecal pellets were weighed to determine the dry weight. The water content of fecal samples was calculated using the following formula:

$$\mathrm{Fecal}\;\mathrm{water}\;\mathrm{content}\;(\%)=\frac{\mathrm{wet}\;\mathrm{weight}\;\left(g\right)-\mathrm{dry}\;\mathrm{weight}\;(g)}{\mathrm{wet}\;\mathrm{weight}\;(g)}\;\times \;100\%$$

#### 4.4.2. Time Determination of First Black Stool

An activated carbon solution was prepared by mixing 100 g of gum arabic (Sinopharm Chemical Reagent Co., Ltd., Shanghai, China) with 800 mL of water, boiling the solution until it became transparent, adding 50 g of activated carbon, then boiling the solution again, and cooling the solution to 15 °C. The solution was diluted to 1000 mL and stored at 4 °C until use. After being fed a charcoal meal, the rats were placed in a dry cage box. The feeding time and time of discharge of the first black stool were recorded, and the time until discharge of the first black stool was calculated.

#### 4.4.3. Determination of Gastrointestinal Mobility Rate

The rats were administered 2 mL of loperamide hydrochloride solution (5 mg/kg bw) by gavage, followed by a charcoal meal after 1 h. After 30 min, the rats were euthanized with pentobarbital sodium (0.5 mL per 100 g bw) and dissected. The total length of the small intestine and the length of the black carbon advance were recorded. The gastrointestinal mobility rate was calculated using the following formula:

$$\mathrm{Small}\;\mathrm{intestine}\;\mathrm{propulsion}\;\mathrm{rate}\;(\%)=\frac{\mathrm{the}\;\mathrm{length}\;\mathrm{of}\;\mathrm{black}\;\mathrm{carbon}\;\mathrm{advance}\;(\mathrm{cm})}{\mathrm{the}\;\mathrm{total}\;\mathrm{length}\;\mathrm{of}\;\mathrm{the}\;\mathrm{small}\;\mathrm{intestine}\;(\mathrm{cm})}\;\times \;100\%$$

### 4.5. Determination of Gastrointestinal Active Peptide

The levels of gastrointestinal active peptides, motilin (MTL, SBJ-R0507), gastrin (GAS, SBJ-R0813), and vasoactive intestinal peptide (VIP, SBJ-R0121) in rat serum were determined using an ELISA kit purchased from Nanjing SenBeiJia Biological Technology Co., Ltd. (Nanjing, China). Specific steps were completed according to the manufacturer’s instructions. To obtain colonic levels of AQP3 (SBJ-R0493) and 5-HT (SBJ-R0128), 20 mg of colonic tissue was weighed and mixed with pre-cooled saline at a mass ratio of 1:9. The mixed samples were homogenized and centrifuged at 12,000× *g* (4 °C) for 15 min. Tissue homogenates were used in the corresponding ELISA kits (Nanjing SenBeiJia Biological Technology Co., Ltd.) to measure AQP3 and 5-HT levels at colonic sites.

### 4.6. Determination of Short-Chain Fatty Acid Content in Feces

The content of SCFAs in feces was determined using gas chromatography–mass spectrometry (GC-MS). Briefly, the fecal samples were diluted with a saturated NaCl solution, acidified with sulfuric acid, extracted with ether, centrifuged, collected, and dehydrated. The ether phase was collected and stored in a gas chromatography injection vial. SCFAs in feces were analyzed using GC-MS, and the parameters were set following those set by Chai [26].

### 4.7. 16S rDNA Sequencing and Bioinformatics Analysis

Total DNA was extracted from rat fecal samples using the Fast DNA Stool Kit (M.P. Biomedicals, Irvine, CA, USA) and used to amplify the V3–V4 regions of the 16S rDNA gene using specific barcoded primers: 341F (5′-CCTACGGGAGGCAGCAG-3′) and 806R (5′-GGACTACHVGGG TWTCTAAT-3′). After the PCR amplification, products were purified by nucleic acid gel electrophoresis, and the PCR products on the gel were collected and purified using a PCR purification kit (TIANgel Mini Purification Kit; TIANGEN, Beijing, China). The concentration of extracted DNA was determined using a NanoDrop 2000c (Thermo Fischer Scientific, Waltham, MA, USA), and the DNA library was constructed by mixing DNA samples at equal concentrations and then sequencing on a MiSeq sequencing machine (Illumina, San Diego, CA, USA).

To examine the composition and organization of the gut microbiota, the operational taxonomic unit (OTU) absolute abundance table was normalized to the total OTU clustering using QIIMEII [56]. Information on specific microbiome species was generated by comparing the sequencing data obtained from OTU cluster analysis with the Silva database. The alpha diversity of the microbial species present in the samples was evaluated to quantify microbial abundance and diversity. Chao1 indices were created to investigate species diversity. The variations in species diversity within the samples were investigated using beta diversity, and individual species differences across samples were calculated using principal coordinate analysis (PCoA). Linear discriminant analysis (LDA) effect size (LEfSe) analysis was used to examine the abundance differences in marker species across groups.

### 4.8. Statistical Analysis

Data are expressed as the mean ± standard deviation (SD). Statistical analyses were performed using GraphPad Prism 8 (version 8.02; GraphPad Inc., San Diego, CA, USA) and IBM SPSS Statistics (version 22.0; SPSS Inc., Chicago, IL, USA). One-way ANOVA with Tukey’s test was used to analyze the differences between each group. *p* < 0.05 indicates statistical significance.

## 5. Conclusions

In this study, the colon-released capsules protected against the release of probiotics with a specific number of viable bacteria in the colon, and the optimal dose of each strain in the colon was explored. Compared with the gastric gavage dose of probiotics, it is even more important to obtain the more appropriate dose in the colon. Daily intake of 6M4 capsules at 10^4^ CFU could effectively relieve constipation caused by loperamide hydrochloride in rats, but the intake thereof at 10^8^ CFU/day may break the balance of intestinal microbes in rats, for which the constipation relief effect is not significant. Of course, the dose–effect relationship varied with different strains. Excessive amounts of certain bacteria are released directly into the colon, which may affect the balance of the gut flora and limit their probiotic effects. In future, we need to develop edible colon-released capsules to encapsulate probiotics. Our results will help correct the confusion of blindly pursuing probiotics with high viable bacterial counts in the market, and enhance everyone’s understanding of the effective dosage of probiotics.

## Figures and Tables

**Table 1:** Viable number of *B. longum* in different groups.

Group	Viable Count	Group	Viable Count	Group	Viable Count
6M4-H	(9.0 ± 0.1) × 10^8^	10M2-H	(7.8 ± 0.2) × 10^8^	S3-H	(4.3 ± 0.1) × 10^8^
6M4-M	(9.8 ± 0.2) × 10^6^	10M2-M	(1.8 ± 0.1) × 10^7^	S3-M	(4.1 ± 0.2) × 10^6^
6M4-L	(2.1 ± 0.1) × 10^4^	10M2-L	(2.3 ± 0.2) × 10^4^	S3-L	(9.7 ± 0.1) × 10^3^
6M4-Free	(1.4 ± 0.3) × 10^9^	10M2-Free	(1.9 ± 0.1) × 10^9^	S3-Free	(5.2 ± 0.3) × 10^8^

The unit of H, M, L groups was CFU/capsule; the unit of free groups was CFU/mL; intragastric volume: one capsule or 2 mL free bacterial suspension per rat per day.

**Table 2:** Grouping of animal experiments and gavage treatment.

Group	Treatment	Count	Group	Treatment	Count
Control	Saline	6	Model	loperamide hydrochloride	6
6M4-H	Capsule (10^8^ CFU) + loperamide hydrochloride	6	6M4-M	capsule (10^6^ CFU) + loperamide hydrochloride	6
6M4-L	Capsule (10^4^ CFU) + loperamide hydrochloride	6	6M4-Free	free (10^8^ CFU/mL) + loperamide hydrochloride	6
10M2-H	Capsule (10^8^ CFU) + loperamide hydrochloride	6	10M2-M	Capsule (10^6^ CFU) + loperamide hydrochloride	6
10M2-L	Capsule (10^4^ CFU) + loperamide hydrochloride	6	10M2-Free	Free (10^8^ CFU/mL) + loperamide hydrochloride	6
S3-H	Capsule (10^8^ CFU) + loperamide hydrochloride	6	S3-M	Capsule (10^6^ CFU) + loperamide hydrochloride	6
S3-M	Capsule (10^4^ CFU) + loperamide hydrochloride	6	S3-Free	Free (10^8^ CFU/mL) + loperamide hydrochloride	6

FGSZY6M4, FJSWXJ10M2, and FSDJN6M3 are abbreviated as 6M4, 10M2, and S3, respectively. H, M, and L refer to high, medium, and low doses, respectively, of *B. longum*; Free means a high number of free *B. longum* suspensions.

## Data Availability

All data presented in this study are available in the main body of the manuscript.
